# Clinical characteristics and treatment evaluation of diffuse large B-cell lymphoma in Chinese children and adolescents: a multicenter clinical study of China-Net childhood lymphoma group B-NHL-2017

**DOI:** 10.1007/s00432-025-06260-4

**Published:** 2025-07-22

**Authors:** Yang Fu, Ling Jin, Yanlong Duan, Jing Yang, Ying Liu, Bo Hu, Mincui Zheng, Yunpeng Dai, Ansheng Liu, Wei Liu, Leping Zhang, Fu Li, Baoxi Zhang, Xiaojun Yuan, Lirong Sun, Rong Liu, Zhuoyu Wen, Runming Jin, Shuquan Zhuang, Lian Jiang, Yufeng Liu, Haixia Zhou, Chen Shen, Hongsheng Wang, Yonghong Zhang, Xiaowen Zhai

**Affiliations:** 1 Department of Hematology, National Children’s Medical Center, Children’s Hospital of Fudan University, Shanghai, 201102 China; 2 Medical Oncology Department, Pediatric Oncology Center, Beijing Children’s Hospital, National Center for Children’s Health, Capital Medical University, Beijing, 100045 China; 3 Department of Pediatric Lymphoma, Beijing Gobroad Boren Hospital, Beijing, 100070 China; 4 Department of Hematology and Oncology, Hunan Children’s Hospital, Changsha, 410007 China; 5 Department of Pediatrics, Shandong Provincial Hospital, Shandong Provincial Hospital Affiliated to Shandong First Medical University, Jinan, 250021 China; 6 Department of Hematology and Oncology, National Children’s Regional Medical Center (Northwest), The Affiliated Children Hospital of Xi’an Jiaotong University, Xi’an Children’s Hospital, Xi’an, 710002 China; 7 Department of Hematology and Oncology, Beijing Children’s Hospital, National Children’s Regional Medical Center, Henan Children’s Hospital, Zhengzhou Children’s Hospital, Zhengzhou Hospital, Children’s Hospital Affiliated of Zhengzhou University, Zhengzhou, 450012 China; 8 Department of Pediatrics, Peking University People’s Hospital, Beijing, 100044 China; 9 Department of Hematology and Oncology, Jinan Children’s Hospital, Children’s Hospital Affiliated to Shandong University, Jinan, 250022 China; 10 Department of Pediatrics, Chinese PLA General Hospital, Beijing, 100039 China; 11 Department of Pediatrics, The Second Hospital of Hebei Medical University, Shijiazhuang, 050004 China; 12 Department of Pediatric Hematology/Oncology, Xinhua Hospital, Shanghai Jiaotong University School of Medicine, Shanghai, 200092 China; 13 Department of Pediatric Hematology and Oncology, The Affiliated Hospital of Qingdao University, Qingdao, 266003 China; 14 Department of Hematology, Capital Institute of Pediatrics, Children’s Hospital, Capital Institute of Pediatrics, Beijing, 100020 China; 15 Department of Pediatric Hematology and Oncology, Northwest Women’s and Children’s Hospital, Shaanxi Maternal and Child Health Hospital, Xi’an, 710061 China; 16 Department of Pediatrics, Union Hospital, Tongji Medical College, Huazhong University of Science and Technology, Wuhan, 430022 China; 17 Department of Pediatrics, Quanzhou First Hospital, Fujian Medical University, Fujian, Quanzhou, 362002 China; 18 Department of Pediatrics, Hebei Tumor Hospital, The Fourth Hospital of Hebei Medical University, Shijiazhuang, 050011 China; 19 Department of Hematology and Oncology, Children’s Hospital, The First Affiliated Hospital of Zhengzhou University, Zhengzhou, 450052 China; 20 Department of Pediatric Hematology, The Second Affiliated Hospital of Wenzhou Medical University, Yuying Children’s Hospital of Wenzhou Medical University, Wenzhou, 325027 China

**Keywords:** Diffuse large B-cell lymphoma, Child, Treatment, Chemotherapy, Prognosis

## Abstract

**Background:**

China-Net Childhood Lymphoma (CNCL) group B-NHL-2017 study is a prospective multi-center study in China, with the purpose of standardizing the diagnosis and treatment of childhood lymphoma, and improving the prognosis.

**Methods:**

From May 2017 to June 2023, 20 centers participated in the diffuse large B-cell lymphoma (DLBCL) study. The clinical data were analyzed to summarize the clinical characteristics, treatment response and outcome. The primary endpoint was 5-year event-free survival (EFS). The trial is registered with the Chinese Clinical Trial Registry (ChiCTR1800020067).

**Results:**

A total of 138 children and adolescents were enrolled, including 101 males and 37 females. The median age of disease diagnosis was 9.0 years (range: 2.3–15.5 years). The range of follow-up time was 17 d–6.0 years. A total of 12 events occurred in this study, including 7 deaths. of which 4 patients died of disease and chemotherapy comorbidities (severe infection, septic shock, etc.), 1 died of disease progression (enlargement of the primary tumor and tumor metastasis), 1 died of recurrence, and 1 died of severe pneumonia in the third year after completing all chemotherapy courses. Recurrence occurred in 6 (4.3%) patients at 14.9 months (range: 4.4–32.6 months) after initial treatment. The 5-year overall survival (OS) was 90.7 ± 5.0% and the 5-year EFS was 85.5 ± 5.4%. Based on Cox regression analysis, no Rituximab during treatment is an independent risk factor for mortality in patients with DLBCL.

**Conclusion:**

The efficacy of CNCL-B-NHL-2017 protocol in the treatment of DLBCL in children and adolescents is close to results of international studies.

## Introduction

Non-Hodgkin lymphoma (NHL) is one of the most common malignant tumors in children and adolescents, and its incidence rate in China ranks third among childhood malignancies, after leukemia and central nervous system tumors (Bao et al. 2016). Diffuse large B cell lymphoma (DLBCL) is one of the subtypes of mature B-NHL, accounting for about 10% of NHL in children (Burkhardt et al. 2005). In recent decades, great progress has been made in the treatment of B-NHL in children and adolescents. Modern chemotherapy protocols can achieve nearly 90% of the event free survival (EFS) at 5 years (Cairo et al. 2007; Minard-Colin et al. 2020; Patte et al. 2001, 2007; Reitere al. 1999; Woessmann et al. 2005). China is a developing country with a large territory and regional gaps in healthcare exists in various places. To establish evidence-based diagnostic and therapeutic guidelines for pediatric lymphoma and enhance clinical outcomes nationwide, the China-Net Childhood Lymphoma (CNCL) collaborative group was initiated in May 2017 as a multicenter registry study. This nationwide consortium encompasses 30 + tertiary medical centers, with its prospectively maintained database capturing epidemiological and treatment data from lymphoma patients across all Chinese provinces and autonomous regions. The CNCL-B-NHL-2017 protocol, based on the LMB89/96 protocol, was implemented as first-line therapy for treatment-naïve pediatric and adolescent patients with histologically confirmed mature B-cell non-Hodgkin lymphoma, encompassing three histopathological subtypes per WHO classification: Burkitt lymphoma, diffuse large B-cell lymphoma (DLBCL), and follicular lymphoma. (Cairo et al. 2007; Patte et al. 2001, 2007). This investigation conducted a comprehensive analysis of all DLBCL cases within the CNCL registry, aiming to elucidate clinicopathological profiles, therapeutic responses, and survival determinants in this understudied population.

## Methods

### Patients

Twenty pediatric cancer centers in North, East, Central and Northwest China participated in the study. Children and adolescents under 16-year-old who were hospitalized in participating centers from May 2017 to March 2023 and were pathologically diagnosed with DLBCL and treated with CNCL-B-NHL-2017 protocol were included. Patients diagnosed with primary mediastinal large B-cell lymphoma (PMBCL) were excluded. This study was registered on the Chinese Clinical Trial Registry, a primary registry of the International Clinical Trial Registry Platform, World Health Organization (No. ChiCTR1800020067).

## Diagnosis and staging

All patients were diagnosed by pathology using immunohistochemistry of tissue biopsy, bone marrow and/or body fluids cytological examination. The pathological diagnosis was made independently by at least two pathologists from tertiary hospitals in accordance to the 2016 revision of the World Health Organization classification of lymphoid neoplasms (Swerdlow et al. 2017). Our protocol stipulates that the same patient’s pathological specimens must undergo consultation by the pathology departments of three tertiary hospitals to ensure diagnostic accuracy. The germinal center B-cell-like (GCB) subtype was defined as the identification of CD10^+^ or CD10^–^/BCL6^+^/MUM-1– tumor cells (Hans et al. 2004; Oschlies et al. 2006). The severity of lymphoma is categorized by revised international pediatric non-Hodgkin lymphoma staging system (IPNHLSS) (Rosolen et al. 2015). CNS infiltration is divided into three levels according to cerebrospinal fluid (CSF) cytology (CSF cell count and cell morphology), clinical manifestations and central nervous system (CNS) imaging examination. CNS1: both imaging and CSF examination were normal. CNS2: meet any one of the following: (a) white blood cell (WBC) > 5/µL in CSF from non-traumatic lumbar puncture and suspicious tumor cells were detected; (b) detectable tumor cells by flow cytometry in negative cytology CSF; (c) traumatic lumbar puncture at diagnosis in patients with bone marrow infiltration; (d) tumor mass identified in head and face, nasopharynx, skull, orbital area, or other sites near the central nervous system. CNS3: meet any one of the following: (a) WBC in CSF > 5/µL with confirmed presence of tumor cells; (b) imaging examination shows space occupying tumor lesion in intracranial area; (c) identified space occupying lesions of the spinal cord.

## Chemotherapy

**Fig. 1:**
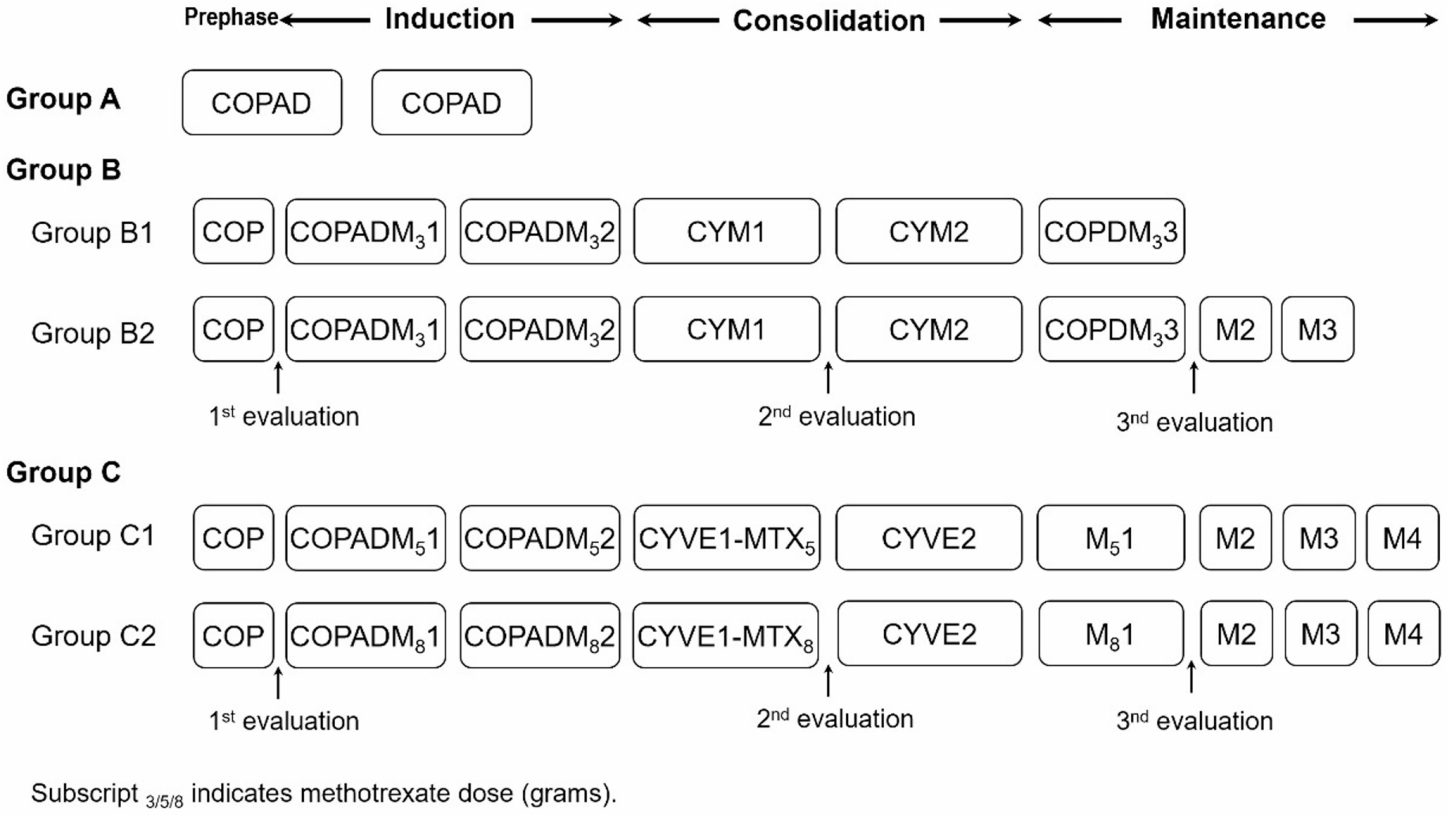
CNCL-B-NHL-2017 mature B-cell lymphoma protocol flow chart

The flow chart and medications of CNCL-B-NHL 2017 chemotherapy protocol are shown in Fig. 1; Table 1. Briefly, the chemotherapy starts with an initial treatment involving a stratified strength of treatment that increasing with disease severity. The patients with DLBCL were triaged into three treatment groups with increased disease severity (A, B and C) according to disease stages and risk factors, including central nervous system (CNS) status. Group A: patients with resected stage I and stage II tumors; Group B: patients with unresected stages I and II or stage III and IV tumors without CNS involvement and blasts in bone marrow < 25%. Group B patients were further divided into Groups B1 and B2 according to the 1st evaluation results on the 7th day (D7) after chemotherapy (Group B1: tumors reduction > 75%; Group B2: tumors reduction 25–75%); Group C: patients with huge tumor mass (single tumor diameter > 10 cm or ≥ 4 organs involvement) or with central nervous system involvement (CNS2 or CNS3), testicular, ovarian and/or bone marrow (blasts > 25%) involvement in stage III and IV tumors. Group C patients were further divided into C1 and C2 groups according to CNS involvement (Group C1: without CNS3; Group C2: with CNS3). In the initial design of this study, rituximab was not included. However, as international research highlighted the excellent efficacy of rituximab treatment, each center was allowed to implement rituximab therapy based on local conditions. (once a day before each chemotherapy course, with a dose of 375mg/m^2^, for a total of 4–6 courses)

**Table 1:** Drug dosage and usage of CNCL-B-NHL-2017 mature B-cell lymphoma protocol

Regimen/Drug	Dose	Administration	Days
COP			
Cyclophosphamide	300 mg/m^2^	IV 15 min	d1
Vincristine	1 mg/m^2^	IV (max. 2 mg)	d1
Prednisone	60 mg/m^2^	orally (in 2 fractions)	d1-7
Methotrexate	15 mg^*^	IT	d1, d3^†^, d5^†^
Cytarabine^†^	30 mg^*^	IT	d1, d3, d5
Dexamethasone	4 mg^*^	IT	d1, d3^†^, d5^†^
COPAD			
Cyclophosphamide	250 mg/m^2^	IV 15 min, q12h	d1-3
Vincristine	2 mg/m^2^	IV (max. 2 mg)	d1, d6
Prednisone	60 mg/m^2^	orally (in 2 fractions)	d1-5, then reduce over 3 d to 0
Daunorubicin	30 mg/m^2^	IV 6 h	d1-2
COPADM_3/5/8_			
Cyclophosphamide^¶^	250 mg/m^2^ 500 mg/m^2‡^	IV 15 min, q12h	d2-4
Vincristine	2 mg/m^2^	IV (max. 2 mg)	d1
Prednisone	60 mg/m^2^	orally (in 2 fractions)	d1-5, then reduce over 3 d to 0
Daunorubicin	30 mg/m^2^	IV 6 h	d2-3
Methotrexate^§^	3 g/m^2^ (for COPADM_3_) 5 g/m^2^ (for COPADM_5_) 8 g/m^2^ (for COPADM_8_)	IV 3 h (group C: 4 h)	d1
Methotrexate	15 mg^*^	IT	d2, d4^†^, d6
Cytarabine^†^	30 mg^*^	IT	d2, d4, d6
Dexamethasone	4 mg^*^	IT	d2, d4^†^, d6
CYM			
Cytarabine	100 mg/m^2^	IV 24 h	d2-6
Methotrexate^§^	3 g/m^2^	IV 3 h	d1
Methotrexate	15 mg^*^	IT	d2
Cytarabine	30 mg^*^	IT	d7
Dexamethasone	4 mg^*^	IT	d2, d7
CYVE			
Cytarabine	50 mg/m^2^	CI 12 h	d1-5
High dose Cytarabine	3000 mg/m^2^	IV 3 h	d2-5
Etoposide	200 mg/m^2^	IV 2 h	d2-5
CYVE-MTX_5/8_ similar to CYVE, plus:			
Methotrexate^§^	5 g/m^2^ (for MTX_5_) 8 g/m^2^ (for MTX_8_)	IV 4 h	d18
Methotrexate	15 mg^*^	IT	d19
Cytarabine	30 mg^*^	IT	d19
Dexamethasone	4 mg^*^	IT	d19
COPDM_3_			
Cyclophosphamide^¶^	500 mg/m^2^	IV 30 min, q12h	d2-4
Vincristine	2 mg/m^2^	IV (max. 2 mg)	d1
Prednisone	60 mg/m^2^	orally (in 2 fractions)	d1-5, then reduce over 3 d to 0
Epirubicin	30 mg/m^2^	IV 6 h	d2-3
Methotrexate^§^	3 g/m^2^	IV 3 h	d1
Methotrexate	15 mg^*^	IT	d2
Dexamethasone	4 mg^*^	IT	d2
M_5/8_1			
Vincristine	2 mg/m^2^	IV (max. 2 mg)	d1
Prednisone	60 mg/m^2^	orally (in 2 fractions)	d1-5, then reduce over 3 d to 0
Methotrexate^§^	5 g/m^2^ (for M_5_1) 8 g/m^2^ (for M_8_1)	IV 4 h	d1
Cyclophosphamide^¶^	500 mg/m^2^	IV 15 min	d2-3
Epirubicin	30 mg/m^2^	IV 6 h	d2-3
Methotrexate	15 mg^*^	IT	d2
Cytarabine	30 mg^*^	IT	d2
Dexamethasone	4 mg^*^	IT	d2
M2			
Cytarabine	50 mg/m^2^	IV, q12h	d1-5
Etoposide	150 mg/m^2^	IV 90 min	d1-3
M3			
Vincristine	2 mg/m^2^	IV (max. 2 mg)	d1
Prednisone	60 mg/m^2^	orally (in 2 fractions)	d1-5, then reduce over 3 d to 0
Cyclophosphamide^¶^	500 mg/m^2^	IV 30 min	d1-2
Epirubicin	30 mg/m^2^	IV 6 h	d1-2
M4			
Cytarabine	50 mg/m^2^	IV, q12h	d1-5
Etoposide	150 mg/m^2^	IV 90 min	d1-3

Subscript _3/5/8_ indicates methotrexate dose (grams). *IV* intravenous, *IT* intrathecal, *CI* continuous infusion.

^*^ IT doses are age adjusted below age 3 years.

^†^ Only for Group C.

^‡^ Only for the second COPADM_5/8_ course (COPADM_5/8_2).

^§^ With citrovorum factor (leucovorin) rescue 24 h after the beginning of methotrexate: 15 mg/m^2^, orally, every 6 h, a total of 12 times. The follow-up is based on the concentration of methotrexate and simultaneous hydration shall be given.

^¶^ If the single dose of cyclophosphamide is up to 500 mg/m^2^, mesna shall be given as a single dose of 200 mg/m^2^ at 0, 4 and 8 h after the start of cyclophosphamide, except hydration.

## Evaluation for therapeutic efficacy

The response to chemotherapy was defined by the 1st evaluation (D7). The reduction of tumor size > 25% at D7 evaluation is defined as sensitive, and ≤ 25% is defined as insensitive. In the 2nd and 3rd evaluation (see Fig. 1).

Treatment intensity was adjusted according to disease evaluation during the therapy. Patients in Group A with insensitive response to therapy on D7 evaluation were upgraded to Group B. Patients in Group B with insensitive response to therapy on D7 evaluation or residual tumor in the 2nd evaluation were upgraded to Group C.

## Definition of event and severe complications

In this investigation, events were operationally defined as tumor progression, disease recurrence, and death during follow-up. It is noteworthy that death may co-occur with other types of events in the same patient. For analytical rigor, these overlapping events were analyzed as composite endpoints with mortality prioritized in statistical analyses according to established oncological research standards.

During the treatment, the following severe complications are recorded: tumor lysis syndrome, posterior leukoencephalopathy syndrome, severe pneumonia, sepsis, severe organ function damage, respiratory and circulatory failure, coma, intracranial hemorrhage and other serious clinical manifestations requiring intensive care.

### Follow up

Patient follow-up was conducted every 3 months during the first and second year after the completion of all chemotherapy. The follow-up is composed of tumor imaging (B-ultrasound, CT/MRI) and blood biochemistry. A comprehensive evaluation is conducted every 6 months, including tumor imaging evaluation, blood biochemistry, immunoglobulin and bone marrow puncture (patients with bone marrow invasion). The patients were then followed up every 6 months from 3 years after chemotherapy. The last follow-up time was April 30, 2023. The EFS was calculated based on the time from diagnosis to the occurrence of outcome events, including disease progression, recurrence, death from any cause, secondary tumor and abandonment of treatment.

## Modifications of CNCL-B-NHL-2017 protocol compared with LMB89/96 protocol

The CNCL-B-NHL-2017 protocol was developed as a modification of the LMB89/96 regimen. Key modifications and their rationale are as follows:​​.

1. Omission of radiotherapy for extramedullary disease:​​ Prior studies indicated that radiotherapy for extramedullary involvement in pediatric NHL did not significantly improve prognosis, yet contributed to cumulative toxicities (Link et al. 1990). ​Accordingly, radiotherapy for extramedullary involvement was omitted in our protocol; instead, intrathecal therapy was intensified.​​
2. Modification of daunorubicin administration:​​ Evidence suggests that reducing the infusion rate of daunorubicin significantly decreases associated toxicities (Cairo et al. 2007). ​Therefore, we decreased the speed of continuous intravenous daunorubicin infusion (60 mg/m2 for 48 h in LMB89/96 protocol was changed to 30 mg/m2 for 6 h × 2 days)​​.
3. ​Risk-stratified subgroup modifications:​​ Patients in Groups B and C were further stratified into subgroups based on distinct risk factors.

​Group B2:​​ Received prolonged chemotherapy to evaluate whether this approach improves clinical outcomes.

​Group C1:​​ Received a reduced chemotherapy dose (high-dose methotrexate decreased from 8 g/m^2^ to 5 g/m^2^) to assess whether toxicity could be mitigated without compromising efficacy.

## Outcomes

The primary endpoint was EFS. Secondary endpoints included any recurrence and overall survival (OS). EFS was computed from the date of diagnosis to the first occurrence of a major adverse event, as defined previously. OS was calculated as the time from diagnosis to death from any cause. In cases where no event occurred, the time to event was censored at the date of the last patient contact.

### Data collection and statistical methods

Researchers from each participating center collect the clinical data of patients into a centralized database platform. This study regularly organizes collaborative group meetings, and regular cross-checking to ensure the data quality by experts of different center in the collaborative group.

Stata 16.0 software (Stata Corp., Texas, TX) was used for statistical analysis, independent sample *t*-test was used for comparing normal distributed continuous variable, and categorical data were compared used *χ*^2^ test. Kaplan-Meier method was used to calculate OS and EFS, and log-rank test was used to compare survival between groups. Using the Cox regression model to analyze independent risk factors for mortality. When the *P*-value is less than 0.05, the difference is considered statistically significant.

## Results

### General information of patients

A total of 138 patients with DLBCL were included in this study. The median age at disease diagnosis was 9.0 (range: 2.3 to 15.5) years old. There were 101 males and 37 females, with a male to female ratio of 2.7:1. Among the sites of biopsy, there were 46 cases of intestinal tissue, 31 cases of superficial lymph nodes or masses, 25 cases of nasopharyngeal tonsils, 19 cases of abdominal mass, 4 cases of hydrothorax or ascites, 4 cases of liver, 3 cases of bone marrow, 2 cases of bone, 1 case of brain tissue, 1 case of gastric tissue, 1 case of spleen and 1 case of testis.

### Staging, CNS involvement and GCB pathological type

Among 138 patients with DLBCL, there were 7 cases (5.0%) in IPNHLSS stage I, 31 cases (22.5%) in stage II, 78 cases (56.5%) in stage III and 22 cases (15.9%) in stage IV. In addition, there were 28 patients (20.3%) with CNS involvement (including 25 cases of CNS2 and 3 cases of CNS3). A total of 120 patients can be classified as GCB lymphoma according to available immunohistochemistry (IHC) (18 cases did not reported complete pathological data for GCB typing), including 84 patients (70.0%) from GCB subtype. In the GCB subgroup, 83 cases were CD10^+^, 1 case was CD10^−^/BCL-6^+^/MUM-1^−^. Among the 84 patients with GCB subtype, 19 cases also expressed BCL-2. There were 36 patients (30.0%) from non-GCB subtype, including 10 cases of CD10^−^/BCL-6^−^ and 26 cases of CD10^−^/BCL-6^+^/MUM-1^+^.

### Events

A total of 12 events were recorded in this study, including 7 deaths. Of these fatalities, 4 were directly linked to disease progression and chemotherapy-related complications, including post-chemotherapy myelosuppression, severe infections, shock, disseminated intravascular coagulation, and multi-organ failure. One patient with mediastinal involvement achieved complete remission (CR) but experienced tumor recurrence at the primary site 6 months post-diagnosis; treatment was discontinued at the family’s request, ultimately resulting in death. Another patient demonstrated a > 75% reduction in tumor size at the D7 evaluation but exhibited disease progression during the third assessment, characterized by primary site tumor enlargement and metastasis to additional locations, leading to death. The final patient successfully completed all prescribed chemotherapy cycles and maintained sustained CR during follow-up; however, they succumbed to severe pneumonia three years after treatment completion.

Furthermore, 5 non-fatal events were documented, all of which were disease relapses. Among these, 3 patients experienced abdominal relapses: one patient achieved long-term survival following hematopoietic stem cell transplantation, another patient remained disease-free after intensification of chemotherapy and radiotherapy, while the third patient was lost to follow-up after relapse. One patient with bone relapse attained long-term survival after CAR-T therapy. Another patient with cervical lymph node relapse declined further treatment but remained alive until the study’s follow-up cutoff date. Including one case of mediastinal relapse that resulted in death, a total of 6 patients experienced tumor recurrence in this study, with a median recurrence time of 14.9 months (range: 4.4 to 32.6 months) following initial chemotherapy.

### Usage of rituximab

Among the 138 enrolled patients in this cohort study, 106 cases (76.8%) received rituximab-containing regimens, while the remaining 32 patients (23.2%) were excluded from rituximab therapy due to multifactorial considerations including medication accessibility constraints, health insurance reimbursement limitations, and patient-driven treatment decisions. Comparative analysis revealed no statistically significant intergroup differences in baseline demographic characteristics, laboratory parameters, or clinical staging (*P* > 0.05 for all comparisons). Notably, the non-randomized treatment allocation and potential confounding factors inherent in this observational design limit direct comparability between the rituximab and non-rituximab cohorts. These findings underscore the necessity for prospective randomized controlled trials to establish evidence-based protocols for optimizing rituximab administration strategies in this patient population.

### Survival status and risk factors

**Fig. 2:**
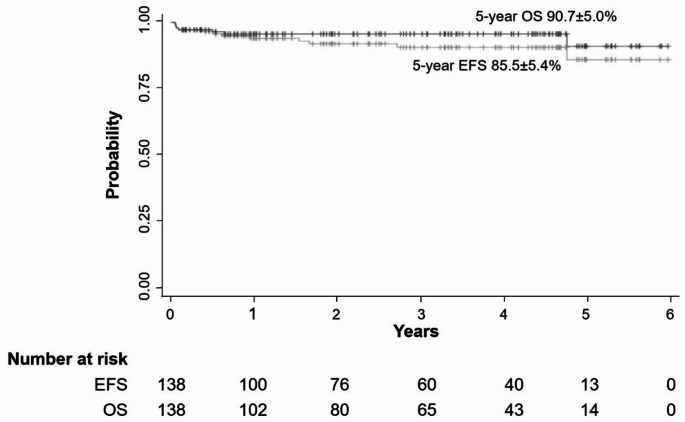
The event free survival (EFS) and overall survival (OS) of DLBCL patients treated with CNCL-B-NHL-2017 protocol

**Fig. 3:**
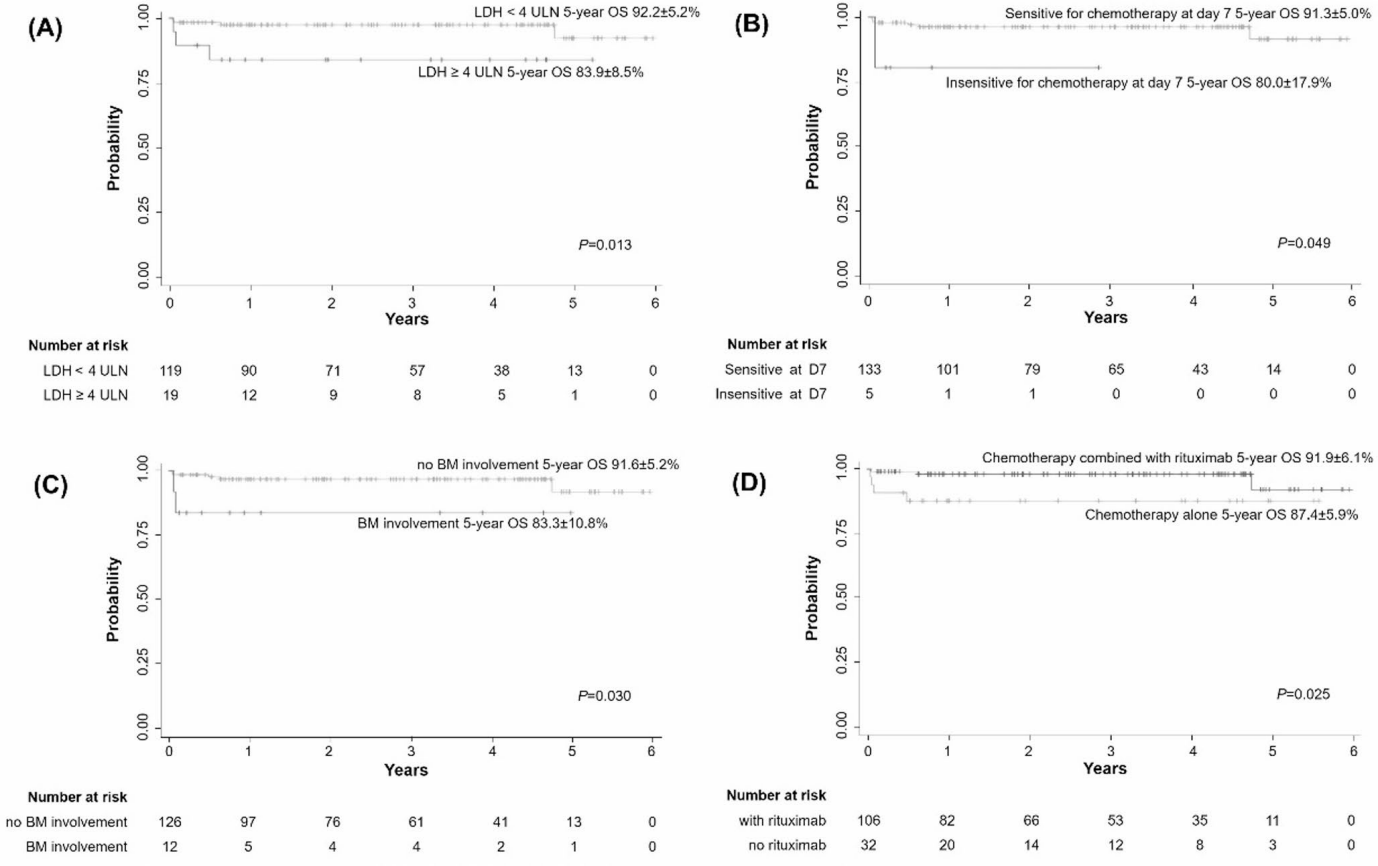
Comparison of overall survival of different subgroups of DLBCL in children treated with CNCL-B-NHL-2017 protocol.** A** the overall survival (OS) of LDH < 4 upper limit of normal (ULN) versus LDH ≥ 4 ULN.** B** OS of sensitive for chemotherapy at day 7 versus insensitive for chemotherapy at day 7.** C** OS of patients with bone marrow (BM) involvement versus without BM involvement.** D** OS of patients treated with chemotherapy alone versus chemotherapy combined with rituximab

The range of median follow-up time was 17 days to 6.0 years. The 5-year OS was 90.7 ± 5.0%, and the EFS was 85.5 ± 5.4% (Fig. 2). Differences in OS and EFS between subgroups are shown in Table 2. Patients with the following factors are associated with increased mortality: (1) lactate dehydrogenase (LDH) > 4 times the upper limit of normal (ULN) at disease diagnosis, (2) insensitive to chemotherapy at D7 evaluation, (3) bone marrow infiltration, (4) no rituximab treatment. Compared with patients without the above factors, the OS was 83.9 ± 8.5% vs. 92.2 ± 5.2%, 80.0 ± 17.9% vs. 91.3 ± 5.0%, 83.3 ± 10.8% vs. 91.6 ± 5.2% and 87.4 ± 5.9% vs. 91.9 ± 6.1%, respectively (*P* < 0.05, Fig. 3). Based on Cox regression analysis, no rituximab during treatment is an independent risk factor for mortality in patients with DLBCL, and the Hazard Ratio was 8.76 (*P* < 0.05) (Table 3).

**Table 2:** Survival rate of diffuse large B-cell lymphoma treated with CNCL-B-NHL-2017 protocol

	*n*	OS (%, ‾x ± s)	χ^2^	*P*-value	EFS (%,‾x ± s)	χ^2^		*P*-value
Gender			2.60	0.107		2.28		0.13
Male	101	87.6 ± 6.5			81.9 ± 7.0			
Female	37	100			96.2 ± 3.8			
Age (years)			0.08	0.96		1.16		0.56
≤ 5	19	94.7 ± 5.1			75.8 ± 17.4			
5–10	64	87.1 ± 9.4			85.7 ± 9.4			
≥ 10	55	94.3 ± 3.2			86.6 ± 5.2			
LDH			6.24	0.013		1.81		0.18
< 4ULN	119	92.2 ± 5.2			86.2 ± 5.7			
≥ 4ULN	19	83.9 ± 8.5			83.8 ± 8.5			
Stage			2.84	0.092		4.92		0.027
I + II	38	100			100			
III + IV	100	86.6 ± 7.3			79.4.0 ± 7.8			
CNS			0.12	0.73		0.95		0.33
Not involvement	110	89.7 ± 5.8			83.3 ± 6.3			
Involvement	28	96.4 ± 3.5			96.4 ± 3.5			
Day 7 evaluation			3.87	0.049		2.41		0.12
Sensitive	133	91.3 ± 5.0			86.0 ± 5.4			
Insensitive	5	80.0 ± 17.9			80.0 ± 17.9			
BM			4.70	0.03		2.05		0.15
Not involvement	126	91.6 ± 5.2			86.0 ± 5.6			
Involvement	12	83.3 ± 10.8			83.3 ± 10.8			
Rituximab			5.03	0.025		1.04		0.31
No rituximab	32	87.4 ± 5.9			87.4 ± 5.9			
With rituximab	106	91.9 ± 6.1			85.3 ± 6.6			
Pathology			1.28	0.26		0.25		0.61
Non-GCB	36	91.7 ± 4.6			88.0 ± 5.7			
GCB	84	87.8 ± 9.4			80.1 ± 10.1			

*OS* overall survival, *EFS* event free survival, *ULN* upper limit of normal, *GCB* germinal center B-cell–like

**Table 3:** Risk factors of death in children with diffuse large B cell lymphoma

Covariant	Haz. Ratio	Standard error	95% CI	z	*P*
Age	0.89	0.12	0.68–1.17	-0.82	0.42
Stage	3.48	2.78	0.73–16.66	1.56	0.12
CNS involvement	0.52	0.78	0.03–9.77	-0.44	0.66
LDH ≥ 4 ULN	2.39	2.15	0.41–13.92	0.97	0.33
Day 7 evaluation	4.89	7.29	0.26–91.35	1.06	0.29
BM involvement	0.71	0.92	0.06–8.90	-0.27	0.79
No Rituximab	8.76	8.76	1.23–62.22	2.17	0.03

*Haz. Ratio* hazard ratio, *CI* confidence interval, *ULN* upper limit of normal.

## Discussion

Data on DLBCL from China is limited due to its low incidence. The recent literature showed that the age of diagnosis is primarily in 15–19 years old, and patients older than 10 years age comprised 60–80% of all DLBCL cases (Oschlies et al. 2006; Reiter et al. 2008; PDQ Pediatric Treatment Editorial Board. 2024). The proportion of patients with stages III and IV disease was 45% in DLBCL. Bone marrow invasion occurred in 1% of all patients with DLBCL and the level of LDH ≥ 500 U/L can be identified in 14% patients (Reiter et al. 2008). In this cohort, the median age at diagnosis was 9.0 years, with 39.9% of patients aged ≥ 10 years at diagnosis. This distribution pattern may be attributed to the study’s upper age inclusion threshold of 16 years, which was lower than that in the aforementioned study. At the same time, the proportion of stage III and stage IV patients in the current study is 72.5%, bone marrow invasion accounts for 8.7%, and LDH ≥ 500 U/L accounts for 20.3% of our patients. The observed discrepancies with prior multinational cohorts may be attributable to the widespread adoption of advanced imaging modalities (e.g., PET/MRI) and enhanced molecular diagnostic techniques over the past decade.

The CNCL-B-NHL-2017 protocol was developed as a modification of the LMB89/96 regimen. We abandoned the use of extramedullary radiotherapy and intrathecal therapy was intensified. Chemotherapy was prolonged for group B2, while the methotrexate dosage was reduced for group C1. Ultimately, no patients in group B2 experienced events, successfully completed chemotherapy, and achieved survival during follow-up; No patients in group C1 experienced CNS-related progression, relapse, death, or other complications.​ However, the clinical significance of these findings requires validation by further controlled trials.​ Concurrently, we also reduced the infusion rate of continuous intravenous daunorubicin and no associated cardiotoxicity was observed in this study cohort. Finally, it must be emphasized that this study only collected and analyzed data from the DLBCL subgroup, more decisive results may require the publication of prognostic data from a multicenter collaborative group encompassing all children with mature B-cell NHL.​

The prognosis of DLBCL in children and adolescents is better than that in adults in general, partly due to a higher prevalence of GCB among children and adolescents (70–80%) than that (40–50%) in adult DLBCL (Chang et al. 2004; Colomo et al. 2003; Hans et al. 2004; Iqbal et al. 2009; Rosenwald et al. 2002). In this study, the GCB subtype in children and adolescents with DLBCL accounted for 70.0%, which was significantly higher than that of non-GCB type and was lower than that reported in 82.7% of international studies (Oschlies et al. 2006). Nevertheless, there was no significant difference in OS and EFS between patients with GCB subtype and non-GCB subtype in this study. In addition, previous study indicated that BCL-2^+^ GCB-DLBCL in adults was associated with significantly reduced OS and EFS (Iqbal et al. 2011). In our study, the proportion of BCL-2^+^ GCB-DLBCL patients was 19/84 (22.6%), lower than that of adults (30%), and there were no death or event among them. These results indicate that the high proportion of GCB subtypes and the low proportion of BCL-2 expression in GCB subgroup are not the main factors that affect the prognosis of children and adolescents with DLBCL better than that of adults. However, the number of cases in our study may still be too small to detect small differences in clinical outcomes.

The use of rituximab has greatly improved the survival of adult DLBCL (Coiffier et al. 2007) and was included in the standard treatment of adulthood DLBCL (Zelenetz et al. 2023). Retrospective analysis of mature B-cell lymphoma in a single center in China also found that the addition of rituximab to NHL patients with LDH ≥ 500 U/L can improve the EFS of patients (Fu et al. 2019). The international multicenter randomized study of high-risk mature B-NHL in children and adolescents has confirmed that the addition of rituximab to the treatment regimen can significantly improve the EFS of patients (Minard-Colin et al. 2020). In this study, the significant improvement in OS in the subgroup treated with rituximab suggest its efficacy in DLBCL.

In China, chemotherapy for malignant tumors in children and adolescents is primarily concentrated in tertiary hospitals located in provincial capitals. Notably, due to a lack of specialized medical personnel, inadequate critical care capabilities, limited diagnostic expertise, insufficient drug support, and patient mistrust, some first-level hospitals and lower-tier medical institutions struggle to implement high-intensity chemotherapy. Additionally, treatment protocols vary across different hospital levels, and there is a lack of comprehensive summaries regarding past treatment experiences and outcomes. To address these challenges, we have established the CNCL collaboration group, inviting medical institutions from all over the country to participate. Our goal is to develop a standardized NHL treatment plan and promote its adoption nationwide, thereby enhancing the quality of treatment at the grassroots level and evaluating the current state of pediatric and adolescent NHL treatment in China.

Internationally, the best treatment protocols for children’s NHL are BFM and LMB multicenter trials. There is no significant difference in survival status of Burkitt lymphoma, DLBCL and other types of NHL, and the EFS was 88–93% (Patte et al. 2001; Patteet al. 2007; Reiter et al. 1999; Woessmann et al. 2005). In our study, the 5-year OS of patients with DLBCL was 90.7 ± 5.0% and EFS was 85.5 ± 5.4%, which was close to the results of major international centers. The results of current study suggest that CNCL-B-NHL-2017 was effective and feasible in the treatment of children and adolescents with DLBCL. In our study, no rituximab during treatment was an independent risk factors for the death of children and adolescents with DLBCL.

## Conclusion

This study is the first multicenter study on the treatment of pediatric DLBCL in China. The efficacy of CNCL-B-NHL-2017 protocol in the treatment of DLBCL in children and adolescents is similar to results of international studies.

## Data Availability

The datasets used and/or analyzed in the current study are available from the corresponding author upon reasonable request.
